# Telling people to “rely on their reasoning” increases intentions to wear a face covering to slow down COVID‐19 transmission

**DOI:** 10.1002/acp.3793

**Published:** 2021-02-03

**Authors:** Valerio Capraro, Hélène Barcelo

**Affiliations:** ^1^ Economics Department Middlesex University London London UK; ^2^ Mathematical Science Research Institute Berkeley California USA

**Keywords:** COVID‐19, dual process, emotion, face masks, reason

## Abstract

Finding messaging to promote the use of face masks is fundamental during a pandemic. Study 1 (*N* = 399) shows that telling people to “rely on their reasoning” increases intentions to wear a face mask, compared with telling them to “rely on their emotions.” In Study 2 (*N* = 591) we add a baseline. However, the results show only a non‐significant trend. Study 3 reports a well‐powered replication of Study 2 (*N* = 930). In line with Study 1, this study shows that telling people to “rely on their reasoning” increases intentions to wear a face mask, compared to telling them to “rely on their emotions.” Two internal meta‐analyses show that telling people to “rely on their reasoning” increases intentions to wear a face mask compared (1) to telling them to “rely on their emotions” and (2) to the baseline. These findings suggest interventions to promote intentions to wear a face mask.

## 1 INTRODUCTION

The coronavirus disease (COVID‐19) pandemic is one of the greatest health threats of the last century. At the time of writing (January 11, 2021), more than 90 million people have tested positive and more than 1.9 million are dead (Worldometers, 2021) – and these are probably substantial underestimations (Burn‐Murdoch et al., 2020).

The large impact of COVID‐19 is partly due to its transmissibility by asymptomatic people, who often are unaware of their infection, through viral droplets in coughs or sneezes (Bai et al., 2020; Mizumoto et al., 2020; Nishiura et al., 2020). For this reason, epidemiologists and health experts have recommended the use of face coverings, with the aim of minimizing the number of infected droplets spread by asymptomatic people, thereby reducing the risk of infecting others. In line with these experts' suggestions, a study based in Germany found that the use of face masks reduced the daily growth rate of reported infections by approximately 40% (Mitze et al., 2020), whereas a study based in Beijing, China, exploring transmission in families with at least one laboratory confirmed COVID‐19 case found that “face mask use by the primary case and family contacts before the primary case developed symptoms was 79% effective in reducing transmission” (Wang et al., 2020).

Yet, we might expect that people may be reluctant to wear a face covering since it represents a significant change in their habitual behavior. It follows that developing mechanisms that favor the use of face masks is crucial to slow down COVID‐19 transmission and “flatten the curve” of the spread. Several national or local governments have taken the difficult decision of making the use of face coverings mandatory in a number of contexts (Javid, 2020). However, since it is impossible to monitor the behavior of every person, even in places where wearing a face covering is mandatory, explicit laws should be complemented by implicit behavioral “nudges” aimed at directing people's behavior towards desired outcomes. In particular, appeals and messages can be effective at promoting desired behavioral changes, because they reach people both inside their homes, through television and social media, as well as outside their homes, through screens, posters, and megaphones. This raises the important question of which types of messaging are effective in promoting the use of face coverings (Van Bavel et al. 2020).

Little is known about this question. Several papers have explored the effect of appeals and messaging on intentions to engage in COVID‐19 preventive behaviors (Bilancini et al., 2020; Capraro & Barcelo, 2020; Everett et al., 2020; Falco & Zaccagni, 2020; Heffner et al., 2020; Jordan et al., 2020; Lunn et al., 2020; Pfattheicher et al., 2020). However, with the exception of one paper, none of these works explored the effect of messages on intentions to wear a face covering; the only exception is Capraro and Barcelo (2020), which found that telling subjects that the coronavirus (COVID‐19) is a *threat to their community* increases intentions to wear a face covering, relative to the baseline. In the current paper, we contribute to this area of the literature by exploring the effect of telling people to “rely on their reasoning” versus telling people to “rely on emotion” versus the baseline with respect to intentions to wear a face covering. This is an important practical question: if one of these messages has a positive effect, it would offer a simple scalable intervention to promote intentions to wear a face covering.

We report three pre‐registered experiments (total *N* = 1920). The experiments were conducted on a heterogeneous, although not representative, sample of people living in the US and surveyed using Amazon Mechanical Turk (Paolacci et al., 2010). The main results are: combining Studies 1–3, we find that telling people to “rely on their reasoning” increases intentions to wear a face covering relative to telling them to “rely on their emotions”; putting Studies 2 and 3 together, we find that the effect is primarily driven by reasoning, meaning that, compared to the baseline, promoting reasoning and logic significantly increases intentions to wear a face covering, whereas promoting emotions does not significantly change intentions to wear a face covering.

## 2 STUDY 1

### 2.1 Method

#### 2.1.1 Conditions

Participants were randomly assigned to one of two conditions: in the *promoting emotion* condition, they were shown a message highlighting the positive consequences of making decisions based on feelings; in the *promoting reasoning* condition, they were shown a message highlighting the positive consequences of making decisions based on reasoning. These messages were taken from previously published work (Capraro et al., 2019; Caviola & Capraro, 2020; Levine et al., 2018). See Table 1 for the exact messages.

**TABLE 1:** Conditions of the experiment

Condition	Message
Promoting emotion	Sometimes people make decisions by using feelings and relying on their emotions. Other times, people make decisions by using logic and relying on their reasoning. Many people believe that emotions lead to good decision‐making. When we use feelings, rather than logic, we make emotionally satisfying decisions. Please answer the following questions by relying on emotions, rather than reasoning.
Promoting reason	Sometimes people make decisions by using logic and relying on their reasoning. Other times, people make decisions by using feelings and relying on their emotions. Many people believe that reason leads to good decision‐making. When we use logic, rather than feelings, we make rationally satisfying decisions. Please answer the following questions by relying on reasoning, rather than emotions.

*Note*: Between‐subjects random assignment.

#### 2.1.2 Dependent variables

After reading the message, all participants took the following scale.

*Intentions to wear a face covering*. Participants were asked to: “answer the following questions by relying on emotions [reasoning]. When the shelter‐in‐place rules are relaxed, I intend to …

1. Wear a face covering any time I leave home.
2. Wear a face covering any time I am engaged in essential activities and/or work, and there is no substitute for physical distancing and staying at home.
3. Wear a face covering any time I'm around people outside my household.”

All answers were collected using a 10‐line “snap to grid” slider with three labels: “strongly disagree” at the extreme left, “neither agree nor disagree” at the center, “strongly agree” at the extreme right.

#### 2.1.3 Demographics

After the scale, participants were asked the following set of demographic questions: sex, age, race, political views, religiosity, whether they live in an urban area, whether wearing a face covering is mandatory in their county, whether they live in an area where shelter‐in‐place rules apply, whether they previously tested positive, whether they believe they will contract coronavirus and, if so, whether they believe they will recover from it relatively easily. At the end, there was a control question to prevent the potential intrusion of bots.

#### 2.1.4 Pre‐registration

The design, the analysis and the sample size were pre‐registered at: https://osf.io/hfjpw/?view_only=cc5aa039b96d4075a3c834c408091992. For this and for the following studies, we report all measures and conditions.

### 2.2 Results

The experiment was conducted on May 28, 2020. The raw data of this and the following studies may be found at: https://osf.io/hfjpw/?view_only=cc5aa039b96d4075a3c834c408091992. The analysis code can be easily replicated by the reader following the analysis below.

#### 2.2.1 Demographic characteristics of the sample

As pre‐registered, we eliminated from the analysis subjects who did not pass the attention check and, for each multiple IP address or Turk ID, we kept only the first observation and discarded the rest. This meant deleting about 1% of the observations; our main results remain qualitatively similar when including these observations. In doing so, we were left with 399 subjects. A posteriori sensitivity analysis shows that this sample size is sufficient to detect an effect size of d = 0.28, with *power of 0.80 and* with α = 0.05*, two‐tailed*. In Table 2, we report the demographic characteristics of the sample for this and the following studies. We note that the sample is quite heterogeneous, although not representative: males and females are equally represented; the age group 25–54 is overrepresented, whereas the age groups 18–24 and 65+ are underrepresented; Whites are overrepresented, while Blacks or African Americans are underrepresented (Census, 2020).

**TABLE 2:** Demographic characteristics of the overall sample

		Percent			
Demographic		Study 1 (*N* = 399)	Study 2 (*N* = 591)	Study 3 (*N* = 930)	All studies (*N* = 1920)
Gender	Female	50.63	50.59	51.29	50.93
	Male	48.62	49.24	48.38	48.70
	Prefer not to say	0.75	0.17	0.32	0.37
Age	18–24	9.27	7.95	6.77	7.65
	25–34	34.59	35.70	40.32	37.71
	35–44	27.32	29.27	25.37	26.98
	45–54	14.04	16.92	14.30	15.05
	55–64	9.77	6.93	8.82	8.43
	65+	4.51	3.21	4.41	4.11
Race	American Indian or Alaska native	1.00	0.51	0.97	0.83
	Asian	11.02	8.13	9.82	9.56
	Black or African American	6.77	7.11	9.06	7.99
	Native Hawaiian or other Pacific Islander	0	0	0	0
	White	77.19	80.33	75.72	77.49
	Multiracial	3.76	3.89	4.42	4.13

*Note*: Political view goes from 1 = “very left‐leaning” to 7 = “very right‐leaning,” with 4 = “center.” In the table we classified as “center” only those subjects who answered “center.”

#### 2.2.2 The effect of promoting emotion versus reasoning on intentions to wear a face covering

We first build the composite variable “intentions to wear a face covering” by taking the average of its three items (*α*_*emotion*_ = 0.932, *α*_*reason*_ = 0.924). The average intention to wear a face covering when promoting reasoning is *M*_*reason*_ = 7.38 (*SD*_*reason*_ = 3.00); the average intention to wear a face covering when promoting emotion is *M*_*emotion*_ = 6.61 (*SD*_*emotion*_ = 3.24). Wilcoxon rank‐sum shows that the distribution of intentions to wear a face covering when reasoning is promoted is statistically different from the corresponding distribution when emotion is promoted (*z* = 2.366, *p* = .018).

## 3 STUDY 2

Study 1 shows that promoting reasoning versus emotion increases intentions to wear a face covering. However, it is not clear whether it is promoting reasoning that increases intentions to wear a face covering, or the opposite, that is, promoting emotion undermines intentions to wear a face covering, or both. To answer this question, in Study 2 we repeat the experiment by adding a baseline condition. Apart from answering our main question, this presents an opportunity to replicate the results of Study 1 (Open Science Collaboration, 2015).

### 3.1 Method

#### 3.1.1 Conditions

Study 2 is identical to Study 1 with the exception that we added the baseline so participants in Study 2 are randomly divided among three conditions: *promoting emotion*, *baseline*, and *promoting reasoning*. In the baseline condition, participants are not presented with any messaging before taking the “intentions to wear a face covering” scale.

#### 3.1.2 Pre‐registration

The design, the analyses, and the sample size were pre‐registered at: https://osf.io/hfjpw/?view_only=cc5aa039b96d4075a3c834c408091992.

### 3.2 Results

#### 3.2.1 Demographic characteristics of the sample

This experiment was conducted on May 29, 2020. People who participated in the previous study were not allowed to participate in this study. As pre‐registered, we eliminated from the analysis subjects who did not pass the attention check and, for each multiple IP address or Turk ID, we kept only the first observation and discarded any others. This corresponds to deleting about 2% of the observations; our main results remain qualitatively similar when including these observations. In doing so, we were left with 591 subjects. A posteriori sensitivity analysis shows that this sample size is sufficient to detect an effect size of f = 0.13, with α = 0.05 and *power of 0.80*. The demographic characteristics of the sample are reported in Table 2.

#### 3.2.2 The effect of promoting emotion versus reason on intentions to wear a face covering

We first build the composite variable “intentions to wear a face covering” by taking the average of its three items (*α*_*emotion*_ = 0.914, *α*_*baseline*_ = 0.937, *α*_*reason*_ = 0.941). The average intention to wear a face covering when promoting reasoning is *M*_*reason*_ = 6.89 (*SD*_*reason*_ = 3.34); the average intention to wear a face covering in the baseline is *M*_*emotion*_ = 6.71 (*SD*_*emotion*_ = 3.24); the average intention to wear a face covering when promoting emotion is *M*_*emotion*_ = 6.65 (*SD*_*emotion*_ = 3.01). A one‐way ANOVA with Bonferroni correction^1^ reveals that there are no statistically significant differences across conditions (*F*(2,588) = 0.31, *p* = .731).

## 4 STUDY 3

Study 2 finds a non‐significant trend in the same direction as Study 1. One possibility is that Study 1 was a false positive. Another possibility is that Study 2 failed to find an effect for some reason. To clarify this, we conducted a third study with a sample size large enough to detect a small effect of *d* = 0.20 with power 0.80 and alpha = 0.05. This sample size was determined by the a priori power analysis reported in the pre‐registration.

### 4.1 Method

#### 4.1.1 Conditions

Study 3 is identical to Study 2.

#### 4.1.2 Pre‐registration

The design, the analyses, and the sample size were pre‐registered at: https://osf.io/hfjpw/?view_only=cc5aa039b96d4075a3c834c408091992.

### 4.2 Results

#### 4.2.1 Demographic characteristics of the sample

This experiment was conducted on June 1, 2020. People who participated in either of the previous two studies were not allowed to participate in this study. As pre‐registered, we eliminated from the analysis subjects who did not pass the attention check and, for each multiple IP address or Turk ID, we kept only the first observation and discarded the rest. This corresponds to deleting about 7% of the observations; our main results remain qualitatively similar when including these observations. In doing so, we were left with 930 subjects. The demographic characteristics of the sample are reported in Table 2.

#### 4.2.2 The effect of promoting emotion versus reason on intentions to wear a face covering

We first build the composite variable “intentions to wear a face covering” by taking the average of its three items *(α*_*emotion*_ = 0.933, *α*_*baseline*_ = 0.941, *α*_*reason*_ = 0.928). A one‐way ANOVA with Bonferroni correction reveals a statistically significant effect of condition on intentions to wear a face covering (*F*(2,927) = 7.35, *p* < .001). Post‐hoc comparisons show that intentions to wear a face covering when reasoning is promoted (*M* = 7.23, *SD* = 2.97) are significantly higher than intentions to wear a face covering when emotion is promoted (*M* = 6.29, *SD* = 3.10), χ^2^ = 0.935, *p* < .001. By contrast, intentions to wear a face covering in the baseline (*M* = 6.71, *SD* = 3.22) do not appear to be significantly different from intentions to wear a face covering when emotion is promoted (χ^2^ = 0.418, *p* = .283) or when reasoning is promoted (χ^2^ = 0.517, *p* = .121).

### 4.3 Pooling the three studies together

**FIGURE 1:**
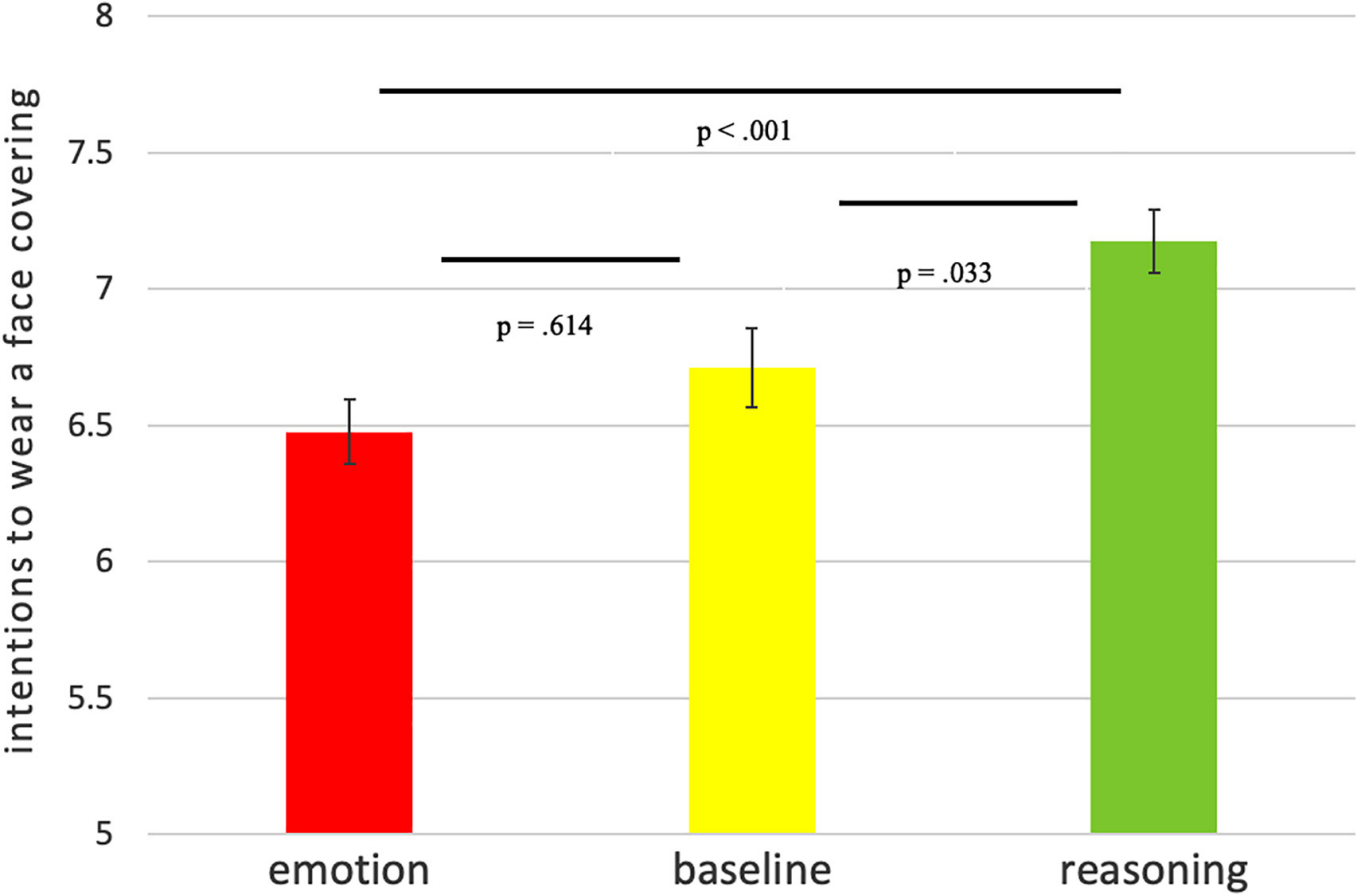
Intentions to wear a face covering split by treatment, all studies together; y‐axis from 0 to 10. Error bars represent the *SE* of the mean. *p*‐values refer to the post‐hoc comparisons after a one‐way ANOVA with Bonferroni correction. Note that the *SEs* do not take into account Bonferroni correction [Color figure can be viewed at wileyonlinelibrary.com]

As pre‐registered in Study 3, we pooled all the data together to increase the power and test which of the three effects are most significant with a larger sample size. Since the three conditions are identical across studies, we simply pooled the data together by condition (Curran & Hussong, 2009). The qualitative results are robust if we instead use random‐effects meta‐analysis.^2^ A one‐way ANOVA with Bonferroni correction confirms the statistically significant effect of condition (*F*(2,1917) = 9.17, *p* < .001). Post‐hoc comparisons reveal that intentions to wear a face covering are higher in the *promoting reason* condition (*M* = 7.18, *SD* = 3.09) compared to the *promoting emotion* condition (*M* = 6.48, *SD* = 3.12), χ^2^ = 0.700, *p* < .001, whereas intentions to wear a face covering in the baseline (*M* = 6.71, *SD* = 3.21) lie between the two other conditions. They are significantly different from the *promoting reason* condition (χ^2^ = 0.466, *p* = .033) but not from the *promoting emotion* condition (χ^2^ = 0.233, *p* = .614). See Figure 1. If we repeat the ANOVA by adding an interaction term condition*study we find that the interaction is not significant (F(3,1916) = 1.00, *p* = .390), suggesting that the effect of condition is similar across studies. Also, the meta‐analysis found no significant heterogeneity across conditions (see Footnote 2).

#### 4.3.1 Exploratory analysis looking at potential moderators of the effect

As exploratory analysis, we added each demographic variable as a separated moderator, in order to test whether the effect of the treatment is particularly strong on any subset of participants. In doing so, we found no significant moderation (sex: F(2,1915) = 0.27, *p* = .896, age: F(106, 1812) = 1.02, *p* = .430; race: F(8, 1906) = 0.80, *p* = .602; political views: F(12, 1905) = 1.39, *p* = .164; religiosity: F(20,1899) = 0.78, *p* = .735; living in an urban area: F(3,1915) = 2.06, *p* = .104; living in a county where wearing a face covering is mandatory: F(4,1915) = 1.38, *p* = .239; living in a county where there are shelter‐in‐place rules: F(4,1915) = 1.32, *p* = .262; tested positive: F(2,1917) = 0.14, *p* = .865; tested negative: F(2,1917) = 0.52, *p* = .594). This suggests that the effect of promoting reason versus emotion is relatively stable across all subsets of the population.

## 5 DISCUSSION

Here we reported three pre‐registered studies exploring the effect of promoting emotion versus reasoning on intentions to wear a face covering. Study 1 shows that telling people to “rely on their reasoning” increases intentions to wear a face mask, compared with telling them to “rely on their emotions.” Study 2 attempts to replicate Study 1, with the addition of a baseline. However, the results show only a non‐significant trend, albeit in the anticipated direction. Study 3 reports a well‐powered replication of Study 2. In line with Study 1, this study shows that telling people to “rely on their reasoning” increases intentions to wear a face mask, compared with telling them to “rely on their emotions.” An internal meta‐analysis shows that telling people to “rely on their reasoning” increases intentions to wear a face mask both compared with telling them to “rely on their emotions” and with the baseline, whereas compared to the baseline, promoting emotion has no effect on intentions to wear a face covering. The latter finding should be taken with caution because the data trend in the direction that promoting emotion decreases intentions to wear a face covering, compared to baseline. Therefore, it is possible that we failed to detect the effect of promoting emotion versus baseline due to insufficient statistical power.

These results contribute to the emerging literature on messaging that increases engagement in preventative COVID‐19 behaviors. Bilancini et al. (2020) found that nudging the personal, or the descriptive, or the injunctive norm has no effect on understanding COVID‐19 related governmental rules. Capraro and Barcelo (2020) reported that telling people that “the coronavirus is a threat to your community” increases intentions to wear a face mask, compared to the baseline. Everett et al. (2020) observed that deontological and virtue‐based messages have little effect on people's intentions to wash their hands, avoid social gatherings, share health messages, and other COVID‐19 preventive behaviors. Falco and Zaccagni (2020) found that reminders which emphasize the consequences of violating social distancing rules on the person him or herself and his or her family increase intentions to engage in social distancing, compared with reminders that emphasize the consequences on other people or the country as a whole. Heffner et al. (2020) reported that threat and prosocial messages increase intentions to self‐isolate. Jordan et al. (2020) observed that showing subjects a flyer with messaging that the coronavirus is a threat to themselves, to their community, or both, increases intentions to engage in several COVID‐19 preventative behaviors, compared to the baseline; Lunn et al. (2020) found that posters focusing on the potential to infect vulnerable people or numerous people are equally effective at increasing caution with respect to social distancing; Pfattheicher et al. (2020) reported that inducing empathy for people most vulnerable to the virus increases intentions to adhere to social distancing and to wear face masks.

These results have practical implications. Finding ways to promote the use of face masks is key during the second phase of the COVID‐19 pandemic response, in which, after the initial strict lockdown, local and national governments are relaxing shelter‐in‐place rules so that some segments of the population are allowed to circulate more freely. Since some of these people will be positive for COVID‐19 without being aware of it, wearing face masks helps to decrease the probability that viral droplets are spread and infect other people. In this light, our results suggest a simple and scalable intervention to promote *intentions* to use face masks: telling people to “rely on reasoning.” This intervention can be scaled up to a national level very simply, for example by sending people text messages with written “Rely on reasoning, rather than feelings: wear your mask.” Similar messages could be shown on national television and social media. A more imaginative way to stage an intervention along these lines is to use alternative messaging such as “Research has shown that wearing a face mask reduces the spread of COVID‐19. Think and wear your mask”! Of course, such interventions would require empirical support.

Of course, our results have some limitations. One regards the sample. Our results were obtained with a heterogeneous, but not representative, sample of people living in the US. We did not find evidence that our results were driven by a particular subset of the population: we included each demographic variable as a potential moderator into separate regression models and found that none of the demographic variables moderated the effect of the messages on intentions to wear a face covering. However, future research should test whether our results can be generalized to the American population at large. Of course, our results cannot be readily generalized to other countries. We suggest that non‐American policymakers who might be interested in using these messages to promote the use of face coverings outside the USA test their effect on intentions to wear a face mask in their countries before implementing them on a large scale. A major limitation of our study is the fact that it focuses on intentions, rather than actual behavior. A recent study found that intentions to practice physical distancing are correlated to actual behavior (Gollwitzer et al., 2020). Although this certainly does not imply that intentions to wear a face covering correlate with actual behavior, it does give some hope that it could actually be the case. Future work should test whether messages of the form used in this paper impact people's actual use of face coverings.

From a theoretical perspective, our results raise the question of why promoting reasoning increases intentions to wear a face covering. There are several possibilities: one is that reasoning deactivates the negative emotions that people feel when wearing a face mask (Capraro & Barcelo, 2020); another is that people generally tend to underestimate their likelihood of infection and reasoning makes them more realistic about their personal health risks (Sjåstad & Baumeister, 2020); another is that reasoning makes people introspect and reflect on their motivations (Wilson & Schooler, 1991; Wilson et al. 1993). Future work could disentangle these potential explanations. Related to these issues, another question is whether the subjects truly acted under emotion/reason or acted *as if* they were under emotion/reason. Disentanglement of these issues could be a worthwhile subject of future research.

## CONFLICT OF INTEREST

The authors declare no conflict of interests.

## Data Availability

The data that support the findings of this study are available at: https://osf.io/hfjpw/?view_only=cc5aa039b96d4075a3c834c408091992.

## Endnotes

^1^
We pre‐registered that we would use pairwise rank‐sum, but then we realized that a one‐way ANOVA with Bonferroni correction would be the correct test to be used in this case. In any case, we note that the pairwise rank‐sum tests, after Bonferroni correction, give qualitatively the same results as the post‐hoc tests of the ANOVA. We make an analogous deviation from the pre‐registration also in Study 3. This is our only deviation from the pre‐registration.

^2^
To analyze the overall effect of promoting emotion versus reasoning, we meta‐analyzed the three effect sizes of Studies 1–3; to analyze the overall effects of promoting emotion versus baseline and promoting reasoning versus baseline, we meta‐analyzed the two effects of Studies 2–3. Since these are pairwise effects, study‐level effects were computed using linear regression. The results were as follows. Regarding the overall effect of promoting emotion versus reasoning, we found an overall effect of 0.344 (95% CI = [0.145, 0.544], *p* = .001), and no evidence of heterogeneity across studies (*p* = .224). Regarding the overall effect of promoting emotion versus baseline, we found an overall effect of 0.277 (95% CI = [−0.113, 0.666], *p* = .164), and no evidence of heterogeneity across studies (*p* = .373). Regarding the overall effect of promoting reasoning versus baseline, we found an overall effect of 0.395 (95% CI = [0.004, 0.786], *p* = .048), and no evidence of heterogeneity across studies (*p* = .426).
